# Case of a Fractured Scull Successfully Treated

**Published:** 1785

**Authors:** 


					II.
Cafe of a fraftured Scull Juccefsfully treated.
By Mr. James Johnfon, Surgeon, at Lane after*
IN February, 1782, I was delired to vifit a
boy in this town who had fallen from a
fcaffold, and was faid to be in a Itate of infen-
fibility with a wound on his head. I found
Bim convulfed, with only a very fmall wound
in the integuments over the middle of the left
parietal bone; but on dilating the wound by a
longitudinal inciiion, and elevating the integu-
ments, I found a portion of the parietal bone,
fomewhat larger than a lhilling, quite loofe.
This I removed with a pair of forceps, and,
after dreffing the wound with dry lint, applied
over
[ 355 1
?ver it a poultice of white bread and milk and
hog's lard.
The convulfions ceafed as foon as the bone
I
was removed, and in a few hours the patient^
was able to walk about as if nothing had hap-
pened. The wound was completely healed in
three weeks ; and, during the cure, he took_no
other medicine than infufion of fena occafionally
to keep his body open.
The fimplicity and fuccefs of the mo\ie of
treatment adopted ia this cafe, will, perhaps,
recommend it to a place in the London Medi-
cal Journal, efpecially as it tends to confirm
the utility of preferving the fcalp in fra&ures
of the fcull, as recommended by Mr. Wilmer
Cafes and Remarks in Surgery.

				

